# Evaluation of 12-Week Standardized Beetroot Extract Supplementation in Older Participants: A Preliminary Study of Human Health Safety

**DOI:** 10.3390/nu16121942

**Published:** 2024-06-19

**Authors:** Vivian dos Santos Pinheiro, Olavo João Frederico Ramos Junior, Caroline Flach Ortmann, Anurag Pande, Carlos Adam Conte-Junior, Thiago Silveira Alvares

**Affiliations:** 1 Nutrition and Exercise Metabolism Research Group, Multidisciplinary Center, Nutrition Institute, Federal University of Rio de Janeiro, Macaé 27971-525, RJ, Brazil; vivianpinheiro14@gmail.com (V.d.S.P.); olavite@gmail.com (O.J.F.R.J.); 2 Postgraduate Program in Food Science, Chemistry Institute, Federal University of Rio de Janeiro, Rio de Janeiro 21941-909, RJ, Brazil; conte@iq.ufrj.br; 3 Multicenter Postgraduate Program in Physiological Sciences, Federal University of Rio de Janeiro, Macaé 27965-045, RJ, Brazil; 4 Sabinsa Brasil Ltd., Florianópolis 88020-302, SC, Brazil; caroline.ortmann@sabinsa.com.br; 5 Sabinsa Corporation, 20 Lake Drive, East Windsor, NJ 08520, USA; anurag@sabinsa.com; 6 Multidisciplinary Center, Food and Nutrition Institute, Federal University of Rio de Janeiro, Macaé 27930-560, RJ, Brazil

**Keywords:** dietary supplements, standardized beetroot extract, nitrate, food safety

## Abstract

In recent years, there has been a notable surge in the popularity of beetroot-based dietary supplements, driven by their rich nitrate composition. Several types of beetroot-based dietary supplements can be found in markets worldwide; however, ensuring the safety of dietary supplements is a crucial consideration, as there is limited evidence on their safety, especially for older populations. Therefore, the purpose of the current study was to evaluate the safety and tolerability of a nitrate-rich beetroot extract in older participants taking supplements over 12 weeks. The participants were randomly assigned to receive 20 g daily of beetroot extract or a matching placebo. The safety and tolerability of the supplementation were evaluated as the occurrence of adverse events and anthropometric, biochemical, and hemodynamic parameters were measured. No serious adverse events were reported in any group. Anthropometric, biochemical, and hemodynamic parameter changes between the baseline and the end of the study were not statistically significant in either group. However, interestingly, the group receiving beetroot extract supplementation exhibited a notable increase in plasma nitrate levels (*p* = 0.076, *f* = 0.50) and showed a decrease in insulin levels (*p* = 0.026, *f* = 0.59). In conclusion, we found that 20 g of beetroot extract supplementation for 12 weeks was safe and well tolerated in older participants.

## 1. Introduction

The senescence process is a determining risk factor for the progress of many cardiovascular diseases, such as atherosclerosis and hypertension, which have a common underlying factor: a progressive decline in endothelial function [1,2]. Vascular endothelial dysfunction occurs during the ageing process and is caused by the deterioration in the equilibrium among vasodilating and vasoconstricting substances made through the endothelium [3]. This imbalance is mainly marked by a progressive reduction in nitric oxide (NO) bioavailability.

In this sense, some nutritional supplements have been proposed to stimulate NO production [4,5]. Among them, beetroot-based supplements have gained prominence as they come from natural sources, which are expected to be safe. Moreover, the increasing popularity of beetroot has demonstrated its enormous market potential as a dietary supplement [6,7]. Beetroot supplements are available as a dietary ingredient in various formulations for distinct health objectives targeting enhancements in cardiovascular health and exercise performance [6,8,9,10]. Beetroot juice is the formulation with the highest nitrate content studied to date and has exhibited favorable outcomes in health, such as reducing blood pressure [5,11]. Moreover, beetroot-based powder formulations have been proven to be advantageous due to their possible health effects and are easy to transport and preserve [12,13]. Thus, the utilization of dietary supplements has dramatically increased among people of all ages, and they are often acquired easily without a prescription or correct guidance on their consumption [14].

Natural dietary supplements are usually associated with health benefits; however, to ensure these benefits are maximized for all, including older populations, safety assessments should be an integral part of their development and studied extensively to recommend their use. Given the possibility that roots, such as beetroot, accumulate heavy metals, such as cadmium (Cd), mercury (Hg), and lead (Pb), prolonged exposure to these may cause some health complications [15,16,17]. For instance, Cd might accumulate this element in the kidneys, leading to nephropathy [18,19]. Furthermore, chronic Pb consumption may lead to neurological disorders (headaches, irritability, depression, seizures, muscle weakness, ataxia, tremors, and hearing impairment), as well as gastroenterological disorders and renal failure [20]. A recent study assessed thirty-seven beetroot-based dietary supplements for their safety and health value based on their mineral composition and barium, aluminum, Cd, and Pb content. The study revealed that five products were considerably contaminated with Cd [6].

Therefore, as part of an ongoing multidisciplinary collaboration to promote the overall safety of beetroot-based dietary supplements, the purpose of the present study was to evaluate the safety and tolerability of supplementation with a nitrate-rich beetroot extract over 12 weeks in subjects aged 60 or older compared to a placebo group.

## 2. Materials and Methods

### 2.1. Participants

**Figure 1:**
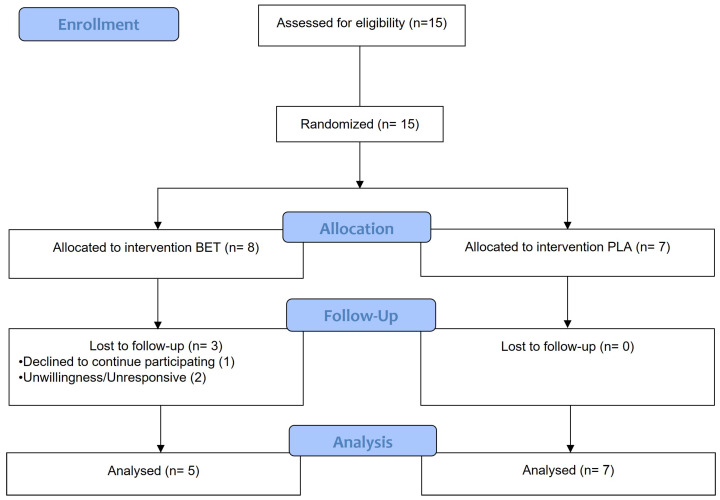
Flowchart of the participant recruitment process and follow-up at 12 weeks.

A total of fifteen participants were assessed for their eligibility. Out of these, twelve older males (3) and females (9), with a mean (±SD) age, height, body mass, and body mass index of 67 ± 6 years, 1.62 ± 0.10 m, 66.0 ± 12.2 kg, and 24.9 ± 3.1 kg/m^2^, respectively, were studied for twelve weeks between June and December 2023 (Figure 1). The baseline characteristics of the participants separated by intervention group can be found in Table 1. All participants were physically active and in good general health, evidenced by their medical history and physical examination. The participants had to meet the following inclusion criteria to participate: being over 60 years old and having a BMI from 22 to 27. The exclusion criteria included being younger than 60, having a severe psychiatric disorder or disease, gastrointestinal, liver, respiratory, or kidney disorder, severe infections such as HIV, uncontrolled diabetes, cancer, or known allergy or intolerance to beetroot. Approval for the study was obtained from the Institutional Ethics Committee of Federal University of Rio de Janeiro, Multidisciplinary Center Macaé, Brazil (protocol CAAE 55245622.2.0000.5699) (10 August 2022), and written, informed consent was obtained from each participant. The trial was registered in the Brazilian Registry of Clinical Trials (ReBEC) with the following identification: RBR-87qh649 (27 March 2023).

### 2.2. Experimental Design

**Figure 2:**
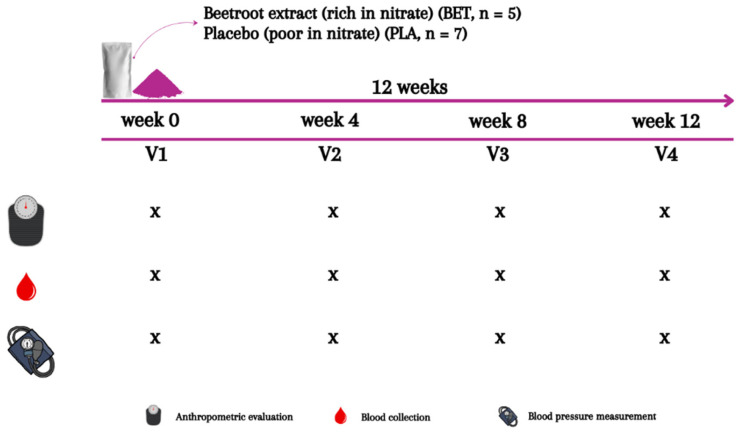
Experimental design.

This was a randomized, double-blind, placebo-controlled, and parallel study with a total duration of 12 weeks. During the study period, the participants reported to the laboratory at Federal University of Rio de Janeiro (Multidisciplinary Center UFRJ-Macaé) on four occasions. Upon the first visit (week 0), anthropometric evaluation was conducted and blood samples and hemodynamic measurements were taken for baseline measurements before supplementation. Afterwards, participants were randomized to treatment (i.e., beetroot extract supplement (BET)) or control (i.e., placebo (PLA)) groups using a free web-based randomization tool (www.randomization.com). Groups A and B were labeled as beetroot extract or placebo by tossing a coin. Beetroot extract supplement and placebo were portioned in pre-packed sachets (equal in weight and similar in appearance) and consecutively numbered for each participant according to the randomization schedule. Each participant was assigned an order number and received the intervention in the corresponding pre-packed sachets. Both the participants and the investigators were blinded to the group allocation. A staff laboratory member who was not involved in the study was responsible for the blinding, group assignment, and allocation concealment. Participants from both BET and PLA groups received the supplements during their first visit, took them home, and were instructed to ingest 10 g of the BET or PLA supplement diluted in 250 mL of water, respectively, twice a day for twelve weeks. Participants received supplements every week and were asked about their adherence to the intervention and possible complications. Anthropometric evaluation was conducted, and blood samples and hemodynamic measurements were taken again in the middle (week 4 and week 8) and at the end of the study period (week 12) (Figure 2). These assessments were carried out at least 12 h after they ingested the second supplement package from the previous day, to avoid acute effects of the supplements. Participants were instructed to maintain their daily routine throughout the study period. Furthermore, participants were asked to fast for at least 12 h (overnight fasting) and refrain from physical exercise for 24 h before each test visit. All visits to the laboratory were held between 08:00 a.m. and 09:00 a.m. At the end of each visit, the participants received a snack consisting of bread and cheese with coffee.

### 2.3. Beetroot Extract

A standardized aqueous beetroot extract (SABEET™, Sabinsa Corporation, East Windsor, NJ, USA) (BET) containing at least 2% nitrate was used in the present study, with the studied batch containing 2.74% nitrate (approximately 548 mg or 8.8 mmol nitrate/day). This amount was chosen based on previous studies demonstrating health benefits after consumption of beetroot supplementation [12,30]. The PLA intervention was prepared by the manufacturer by removing nitrate from the BET. The PLA was indistinguishable from the BET in packaging, color, texture, taste, and smell. The PLA contained a 0.23% nitrate content (approximately 0.7 mmol/20 g), and both supplementations were evaluated in our laboratory using a high-performance liquid chromatography (HPLC) system, as described by a previous study [31]. An approximate nutritional profile of beetroot extract (both supplements) can be found in Table 2.

### 2.4. Anthropometric Measurements

Body mass was assessed using an Omron^®^ bioimpedance scale (model HBF-514C), with participants wearing no shoes and light clothing. A compact tape-based stadiometer (model, branch) was employed to measure body height, with the participants standing upright. BMI from each participant was calculated using their body mass (Kg) and height values (m) and classified according to reference values [21,32].

### 2.5. Biochemical Analysis

Blood samples were drawn from participants’ antecubital veins, collected in a tube with clot activation gel and EDTA, and immediately centrifuged at 6000× *g* at 25 °C for 20 min to separate the serum and plasma. These were taken after overnight fasting at week 0, week 4, week 8, and week 12 to evaluate biomarkers of glucose homeostasis (fasting glucose, insulin, and glycated hemoglobin—HbA1C), lipid profile (triglycerides—TG, total cholesterol—TC, high-density lipoprotein cholesterol—HDL-c, low-density lipoprotein cholesterol—LDL-c, and very-low-density lipoprotein cholesterol—VLDL-c), kidney function (creatinine—Cr and uric acid—UA), liver enzymes (aspartate aminotransferase—AST, alanine aminotransferase—ALT, alkaline phosphatase—ALP, and γ-glutamyl transpeptidase—GGT), and nitric oxide metabolites (nitrate and nitrite).

Fasting glucose and HbA1C levels were measured by enzymatic method; lipid and hepatic parameters (ALP and GGT) were evaluated by enzymatic colorimetric method; and hepatic parameters (AST and ALT) and kidney parameters (Cr and UA) were measured by kinetic method using automatic equipment (Architect c4000). Insulin levels were determined using the chemiluminescence enzyme immunoassay method with automatic equipment (Alinity i). Both analyses were carried out in accordance with the manufacturer’s manual (Abbott) [33,34]. Homeostasis model assessment of insulin resistance (HOMA-IR) values were calculated using the following formula: insulin (µIU/mL) × fasting glucose (mg/dL)/405; and homeostasis model assessment of beta-cell function (HOMA-β) values were calculated using the following formula: (20 × insulin (µIU/mL))/((fasting glucose (mg/dL × 0.0555)/−3.5).

Plasma nitrate analysis was performed as previously described by Croitoru et al. (2012) [31]. Briefly, 150 μL of serum was added into a 1.5 mL microtube with 150 μL of acetonitrile. The mixture was vortex-mixed for 30 s and then centrifuged at 14,000× *g* at 25 °C for 10 min. Subsequently, the supernatant was transferred, 150 μL of mobile phase (5 mM tetrabutylammonium hydroxide (at pH of 2.5) was added, and the mixture was mixed using a vortex mixer for 30 s. Finally, 150 μL was injected into the HPLC system (Model LC—20A Prominence, Shimadzu, Tokyo, Japan), which was fit with LC18 column (15 × 4.6 mm, Sigma-Aldrich, Saint Louis, MO, USA), guard column (5-mm, 50 × 4.6 mm), and photodiode array detector monitoring absorbance to nitrate at 222 nm.

Plasma nitrite analysis was performed, using a process adapted from that of previous studies [35,36]. Briefly, 100 μL of serum was added to 100 μL H_2_O Mili-Q and 600 μL of methanol. The mixture was mixed in a vortex mixer for 30 s and then centrifuged at 10,000× *g* at 25 °C for 10 min. After 200 μL of the supernatant was transferred, 20 μL of 316 µM 2,3-diaminonaphthalene solution was added, mixed in a vortex mixer for 30 s, and placed in a water bath at 24 °C for 10 min. Immediately after, 10 µL of the 2.8 M sodium hydroxide solution was added. This was then mixed in a vortex mixer, and finally, 150 μL was injected into the HPLC system (Prominence, Shimadzu, Tokyo, Japan), which was fit with an LC18 analytical column (150 × 4.6 mm), guard column (50 × 4.6 mm), and fluorescence detector set at 355 nm (excitation) and 460 nm (emission).

### 2.6. Hemodynamic Measurements

The systolic blood pressure (SBP), diastolic blood pressure (DBP), and heart rate (HR) measurements were taken in duplicate on the left brachial artery in a seated position using a validated blood pressure monitor (BP791It, Omron Co., Tokyo, Japan) and appropriate-sized upper-arm cuff after 5 min of rest. Measurements were taken at week 0, week 4, week 8, and week 12.

### 2.7. Statistical Analysis

The assumptions of normality, homogeneity of variances, and sphericity were examined with the Shapiro–Wilk, Levene, and Mauchly tests, respectively. A two-way mixed ANOVA with repeated measures (between/within-subjects design) was used to identify differences in biochemical, anthropometric, and hemodynamic measurements between BET and PLA groups at week 0, week 4, week 8, and week 12. Additional post-hoc tests with Bonferroni adjustment were performed when a significant *F* was found. Furthermore, the area under the curve (AUC) from week 0 to week 12 was calculated, and an independent *t*-test was used to identify differences in the AUC between BET and PLA groups. An *a priori* power analysis was performed using G*Power software (version 3.1). The statistical power was set at 1 − β = 0.80, the effect size was set at *f* = 0.35 (based on previous studies, such as that of Barros-Santos et al. (2020) [37]), and the overall level of significance was set at α = 0.05. At least fifteen participants were required to ensure to a particular degree of certainty that the study had acceptable amount of power to support the null hypothesis. Cohen’s *f* and *d* were calculated as a measurement of the size of the differences between groups (i.e., effect size), and the interpretation was based on the following benchmarks: *f* = 0.1 or *d* = 0.2 indicated small effect; *f* = 0.25 or *d* = 0.5 indicated medium effect; and *f* = 0.4 or *d* = 0.8 indicated large effect [38]. Statistical significance was set at the 0.05 level of confidence. All analyses were performed using a commercially available statistical package (IBM SPSS Statistics version 26 for Mac). The results are expressed as means ± SD. The graphics were designed using GraphPad Prism 9.

## 3. Results

Twelve participants completed the study, demonstrating that the beetroot extract dosage in this research study was well tolerated. Throughout the 12-week supplementation period, no adverse events were observed, except for flu-like symptoms reported by two individuals in the placebo group which were unrelated to the supplementation. The participants from the BET group reported the presence of pink or reddish coloring in their urine and stools, which is common after consuming beetroot and does not indicate a health-related problem. 

**Figure 3:**
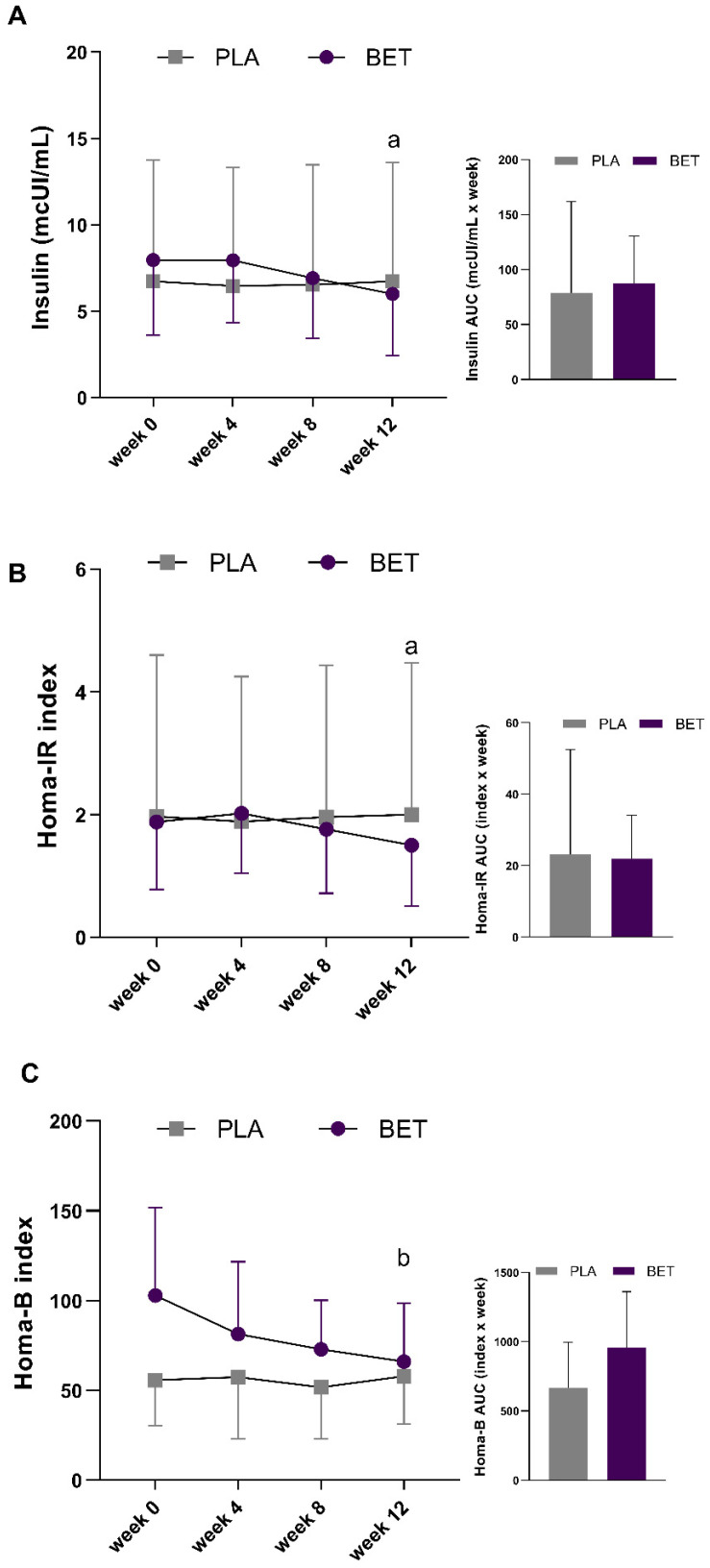
Changes in health biomarkers in the beetroot extract (BET) and placebo (PLA) groups over the 12-week supplementation period. (**A**) Fasting insulin; (**B**) Homeostatic model assessment of insulin resistance index, HOMA-IR; (**C**) Homeostasis model assessment of beta-cell function index, HOMA-β. ^b^ Different (*p* < 0.05) compared to week 0 and ^a^ different (*p* < 0.05) compared to week 4 for the BET group.

Table 3 shows data about the AUC and confidence interval of anthropometric, biochemical, and hemodynamics parameters of both the BET and PLA groups. Regarding biochemical parameters, no significant differences between the BET and PLA groups were observed in terms of their lipid profile for TC (main effect for time, *p* = 0.514, *f* = 0.27; interaction effect, *p* = 0.434, *f* = 0.30), TG (main effect for time, *p* = 0.378, *f* = 0.32; interaction effect, *p* = 0.696, *f* = 0.21), HDL (main effect for time, *p* = 0.397, *f* = 0.32; interaction effect, *p* = 0.294, *f* = 0.36), LDL (main effect for time, *p* = 0.593, *f* = 0.25; interaction effect, *p* = 0.702, *f* = 0.21), or VLDL (main effect for time, *p* =0.352, *f* = 0.33; interaction effect, *p* = 0.724, *f* = 0.20). In terms of their hepatic function markers, no statistical difference between the BET and PLA groups was demonstrated for GGT (main effect for time, *p* = 0.382, *f* = 0.30; interaction effect, *p* = 0.685, *f* = 0.16), AST (main effect for time, *p* = 0.487, *f* = 0.26; interaction effect, *p* = 0.459, *f* = 0.27), ALT (main effect for time, *p* = 0.600, *f* = 0.25; interaction effect, *p* = 0.524, *f* = 0.27), or ALP (main effect for time, *p* = 0.665, *f* = 0.22; interaction effect, *p* = 0.285, *f* = 0.36). In terms of kidney function markers, no statistical difference between the BET and PLA groups was observed for Cr (main effect for time, *p* = 0.938, *f* = 0.05; interaction effect, *p* = 0.665, *f* = 0.18) or UA (main effect for time, *p* = 0.774, *f* = 0.19; interaction effect, *p* = 0.392, *f* = 0.32). Regarding glucose homeostasis, no significant difference was observed in fasting glucose levels between the groups (main effect for time, *p* = 0.132, *f* = 0.44; interaction effect, *p* = 0.457, *f* = 0.29). Likewise, the mean fasting glucose AUC values were not different between the BET and PLA groups (*p* = 0.658, *d* = 0.27). In contrast, a significant interaction effect was observed for insulin levels (*p* = 0.026, *f* = 0.59), with the BET group showing lower levels between week 4 and week 12 (*p* = 0.038), even though there was no significant main effect for time (*p* = 0.050, *f* = 0.53). However, the insulin AUC from week 0 to week 12 was not different statistically between the groups (*p* = 0.841, *d* = 0.12). The HOMA-IR index was also reduced only in the BET group between week 4 and week 12 (*p* = 0.034), and the HOMA-β index showed a significant difference between week 0 and week 12 (*p* = 0.014) (Figure 3) (Table S1).

**Figure 4:**
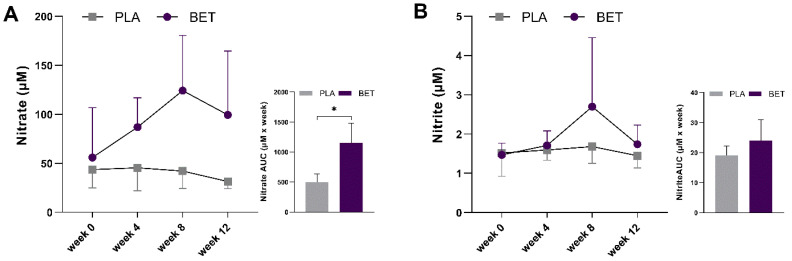
Changes in plasma NO metabolites between beetroot extract (BET) and placebo (PLA) groups over the 12-week supplementation period. (**A**) Nitrate (**B**) Nitrite. * Indicates difference (*p* < 0.05) between groups.

The markers of nitric oxide metabolism, nitrate (main effect for time; *p* = 0.138, *f* = 0.40; interaction effect, *p* = 0.076, *f* = 0.50), and nitrite (main effect for time, *p* = 0.081, *f* = 0.49; interaction effect, *p* = 0.269, *f* = 0.37) were not statistically different. The nitrate_AUC_ significantly increased in the BET group compared to the PLA group (*p* = 0.008, *d* = 2.63). However, there were no significant changes in the nitrite_AUC_ between the groups even though there was a very large effect size (*p* = 0.188, *d* = 0.93) (Figure 4).

No significant changes were observed in SBP (main effect for time, *p* = 0.742, *f* = 0.20; interaction effect, *p* = 0.665, *f* = 0.22), DBP (main effect for time, *p* = 0.364, *f* = 0.33; and interaction effect, *p* = 0.258, *f* = 0.37), or HR (main effect for time, *p* = 0.051, *f* = 0.54; interaction effect, *p* = 0.765, *f* = 0.19) over the 12-week supplementation period for both the BET and PLA groups.

None of the anthropometric measurements were statistically different between the groups during the supplementation period, as there were no changes in body mass (main effect for time, *p* = 0.441, *f* = 0.28; interaction effect, *p* = 0.452, *f* = 0.28) or BMI (main effect for time *p* = 0.448, *f* = 0.27; interaction effect, *p* = 0.347, *f* = 0.33) over time. The body mass_AUC_ (*p* = 0.540, *d* = 0.35) and BMI_AUC_ (*p* = 0.664, *d* = 0.24) also showed no statistical differences. 

## 4. Discussion 

The present study aimed to investigate the safety and tolerability of beetroot-based supplements. Accordingly, we assessed participants’ blood biomarkers as well as hemodynamic and anthropometric variables as health parameters for 12 weeks. The findings of this study showed that most of their blood biomarkers as well as hemodynamic and anthropometric variables were not significantly changed. The findings reveal that the standardized beetroot extract was well tolerated and accepted by the participants and showed a positive impact on their glycemic metabolism markers. This suggests potential health benefits from the use of beetroot extract. However, further long-term studies are necessary to confirm these findings and provide evidence for the use of beetroot extract for glycemic support. 

Interest in the development of beetroot-based products with potential beneficial effects on human health has increased exponentially [6,39,40]. More specifically, beetroot supplements have been recommended to promote cardiovascular health [8], as studies have shown that their intake increases the bioavailability of nitric oxide [41,42], reduces systolic blood pressure [10,43,44], and improves endothelial function in different population groups [45,46,47]. Beetroot extract supplementation can support healthy cardiovascular function in aging populations, as aging has been associated with a reduced bioavailability of nitric oxide [3,48] and a high prevalence of cardiovascular diseases [49]. Given this, innovation in the development of powdered vegetable-based food supplements can contribute to the practicality of ingesting bioactive compounds beneficial to the health of older populations, making it important to evaluate their safety.

Our results have shown that daily supplementation with 20 g standardized beetroot extract for 12 weeks was safe and well tolerated in older participants. No serious adverse events were observed, and minor adverse events (i.e., flu-like symptoms) were unrelated to the consumption of the standardized beetroot extract. Also, no other symptoms that could be associated with the accumulation of heavy metals were related by the participants, which could be justified by the low heavy metal content found in the beetroot extract used in the present study. Only beeturia (a reddish coloring in the urine and stools) was reported by participants, which is caused by the pigmentation of the beetroot. This can be explained by the presence of betalain in the composition of this food, which has potential health benefits, and no association with hepatic changes was demonstrated [50]. These results reinforce those of previous studies showing the safety of beetroot supplementation at similar doses in different adult populations [51,52,53]. In addition, there were no differences between the beetroot extract and control groups in anthropometric and liver and kidney function biomarkers. The data from the present study corroborate a previous study [54], which reported no significant changes in serum creatinine after beetroot intake other than a reduced renal resistive index in patients with chronic kidney disease. Bahadoran et al. (2021) [55] also investigated liver and kidney markers over time after the ingestion of beetroot powder in a population with diabetes, with no changes observed in these biochemical markers (e.g., ALT and UA, respectively) that could be harmful to health, attesting to the safety of ingesting this dietary source of nitrate. 

In line with a previous study [56], a decrease in fasting insulin levels was observed between the start and the end of the present study only in the BET group. Wootton-Beard et al. (2014) [56] observed significantly lower insulin and glucose levels after participants ingested 225 mL of beetroot juice compared with a control group. The authors suggested that the polyphenols (e.g., betalains) and nitrate found in the beetroot may have contributed to the decrease in insulin concentration. In addition to the properties of beetroot that are beneficial for glycemic control, two studies [57,58] have highlighted its effect on lipid profile variables (i.e., TC, LDL-c, HDL-c, and TG), demonstrating reduced concentrations of TC, LDL-c, and TG after a single dose [58] and six weeks [57] of beetroot juice supplementation. The mechanism by which beetroot can lead to changes in lipid metabolism is not entirely understood. However, corroborating the findings of the present study, a recent systematic review with a meta-analysis [59] has demonstrated that beetroot consumption had no significant effect on any lipid profile variables, concluding that beetroot consumption cannot be categorized as an effective nutrition strategy for the adjustment of one’s lipid profile.

In this study, no significant differences were observed in systolic and diastolic blood pressure among the groups throughout the 12-week period of beetroot supplementation. This outcome diverges from the findings of previous research [60,61,62,63,64] which highlighted the potential of beetroot to support cardiovascular health. However, it is important to note that the absence of significant changes in our study does not negate the cardiovascular benefits of beetroot identified in earlier studies [30,65]. The variability in blood pressure responses to dietary nitrate may be influenced by a participant’s initial blood pressure levels [66]. Additionally, the presence of blood pressure medications among some of the older participants could have masked the beetroot extract’s potential beneficial effects on blood pressure regulation.

Altogether, given the preliminary nature of this safety study, the small number of participants resulted in a limited statistical power to demonstrate the specific benefits of the beetroot extract on health-related parameters. Furthermore, given the limited number of male participants (i.e., *n* = 3), a further analysis to determine sex differences in the investigated variables after beetroot extract supplementation was not possible. However, to complement our findings, we have calculated the effect size, since this statistical measurement is not affected by sample size. The effect size provides the practical significance of beetroot extract ingestion (the magnitude of the effect) on the anthropometric, biochemical, and hemodynamic parameters investigated [67,68]. For instance, the participants’ plasma nitrate levels were greater after 12 weeks of beetroot extract supplementation compared to those of the placebo group (*d* = 2.63). Similarly, the participants’ insulin levels were greater after the beetroot extract supplementation compared to those of the placebo group (*f* = 0.59). That being said, the primary aim of the present study was fulfilled, namely, the investigation of the safety and tolerance of standardized beetroot extract supplementation in a limited sample of an older population. Even though the overall response indicates that the beetroot standardized extract used in the present study can be considered safe and was well tolerated, the present findings need to be cautiously interpreted since they still rely on a small number of participants.

## 5. Conclusions

In conclusion, we found that 20 g of standardized beetroot extract supplementation (containing 548 mg or 8.8 mmol nitrate/day) for 12 weeks was safe and well tolerated in older participants. It was necessary to conduct this study before investigating the efficacy of this dietary supplement in a larger sample of older people to detect changes in other health parameters, such as vascular function and muscle quality, and to promote the overall safety and efficacy of beetroot-based dietary supplements.

## Figures and Tables

**Table 1:** Baseline characteristics of the participants.

	BET	PLA	Reference Value [21,22,23,24,25,26,27,28,29]	*p*
Demographics				
N (male)	5 (1)	7 (2)	-	-
Age (years)	64 ± 5	69 ± 5	-	0.111
Anthropometric measurements				
Weight (kg)	68.7 ± 15.8	64.8 ± 9.8	-	0.535
Height (m)	1.64 ± 0.13	1.61 ± 0.07	-	0.611
BMI (kg/m^2^)	25.4 ± 4.4	24.5 ± 2.2	22.00–27.00	0.701
Biochemical analysis				
Glucose (mg/dL)	93.40 ± 17.00	103.28 ± 21.16	<100.00	0.409
Insulin (mcIU/mL)	7.96 ± 4.36	6.73 ± 7.03	<25.00	0.738
HOMA-IR index	1.88 ± 1.10	1.97 ± 2.62	<2.35	0.944
HOMA-B index	102.96 ± 48.75	55.78 ± 25.45	>94.74	0.052
HbA1C	5.54 ± 0.42	5.80 ± 0.42	<5.70	0.505
TG (mg/dL)	102.80 ± 62.43	102.43 ± 50.37	<150.00	0.545
TC (mg/dL)	198.00 ± 21.76	171.14 ± 32.32	<190.00	0.139
HDL-c (mg/dL)	60.20 ± 10.73	62.14 ± 15.50	>40.00	0.815
LDL-c (mg/dL)	116.00 ± 24.30	90.43 ± 32.04	<130.00	0.116
VLDL-c (mg/dL)	22.00 ± 9.80	18.57 ± 9.25	-	0.550
Creatinine (mg/dL)	0.78 ± 0.13	0.81 ± 0.13	0.50–1.30	0.669
Uric acid (mg/dL)	4.38 ± 0.88	4.80 ± 0.53	<7.00	0.324
GGT (IU/L)	28.80 ± 28.08	28.28 ± 11.82	<55.00	0.966
ALP (IU/L)	66.60 ± 15.66	66.28 ± 16.40	<128.00	0.974
AST (IU/L)	30.20 ± 17.02	23.00 ± 6.30	<35.00	0.323
ALT (IU/L)	24.00 ± 16.47	17.71 ± 5.31	<45.00	0.187
Nitrate (µM)	55.90 ± 50.79	43.60 ± 18.79	<59.60	0.565
Nitrite (µM)	1.47 ± 0.30	1.51 ± 0.59	<1.50	0.628
Hemodynamic analysis				
SBP (mmHg)	126.80 ± 6.26	119.85 ± 7.82	<140.00	0.132
DBP (mmHg)	86.00 ± 11.13	74.42 ± 7.52	<90.00	0.056
HR (bpm)	73.80 ± 10.44	64.71 ± 10.62	<80.00	0.172

Abbreviation: ALP, alkaline phosphatase; ALT, alanine transaminase; AST, aspartate transaminase; BMI, body mass index; bpm, beats per minute; DBP, diastolic blood pressure; GGT, γ-glutamyl transpeptidase; HbA1C, glycated hemoglobin; HDL-c, high-density lipoprotein cholesterol; HOMA-B, homeostasis model assessment of beta-cell function; HOMA-IR, homeostatic model assessment of insulin resistance; HR, heart rate; LDL-c, low-density lipoprotein cholesterol; IU/L, international units per liter; kg, kilogram; m, meters; mcIU/dL, micro international unit per milliliter; µM, micromolar; mg/dL, milligrams per deciliter; mmHg, millimeter of mercury; SBP, systolic blood pressure; TC, total cholesterol; TG, triglycerides; VLDL-c, very-low-density lipoprotein cholesterol. No significant difference between groups. Statistical significance was set at the 0.05 level of confidence. Values are expressed as mean ± standard deviation.

**Table 2:** Proximate and chemical composition of the beetroot extract—Sabeet™ (BET and PLA).

Proximate Composition	
Carbohydrates (%) *w*/*w*	80
Sugars (%) *w*/*w*	61
Fat (%) *w*/*w*	0.5
Protein (%) *w*/*w*	6.3
Ash (%) *w*/*w*	5.2
Moisture (%) *w*/*w*	3.5
Mineral	
Calcium (%) *w*/*w*	0.1
Potassium (%) *w*/*w*	1.0
Sodium (%) *w*/*w*	0.7
Iron (%) *w*/*w*	n.d.
Heavy metal	
Lead (µg/g) ppm	<0.2
Arsenic (µg/g) ppm	<0.2
Cadmium (µg/g) ppm	<0.2
Mercury (µg/g) ppm	<0.1

Abbreviation: BET, standardized aqueous beetroot extract rich in nitrate; n.d., not detected; µg/g, micrograms per grams; PLA, standardized aqueous beetroot extract poor in nitrate (placebo); ppm, parts per million; *w*/*w*, weight per weight.

**Table 3:** Area under the curve (AUC), mean difference, and 95% confidence interval for the anthropometric, biochemical, and hemodynamics parameters.

	AUC		Mean Difference	95% Confidence Interval	
	BET	PLA		Lower Limit	Upper Limit
Anthropometric measurements					
Body mass (kg)	829.2 ± 205.2	771.4 ± 110.2	−57.79 ± 90.97	−260.48	144.90
Height (m)	19.33 ± 0.89	19.70 ± 1.53	−0.37 ± 0.70	−1.92	1.19
BMI (kg/m^2^)	305.9 ± 53.3	295.8 ± 24.3	−10.12 ± 22.61	−60.50	40.27
Biochemical analysis					
Glucose (mg/dL)	1196.4 ± 230.3	1271.0 ± 308.3	74.60 ± 163.76	−290.28	439.48
Insulin (mcIU/mL)	87.3 ± 43.1	78.9 ± 82.8	−8.38 ± 40.78	−99.25	82.48
HOMA-IR index	21.9 ± 12.1	23.1 ± 29.4	1.18 ± 14.07	−30.16	32.53
HOMA-B index	955.6 ± 405.7	664.0 ± 332.0	−291.55 ± 212.71	−765.50	182.40
TG (mg/dL)	1247.6 ± 386.0	1253.9 ± 637.1	6.26 ± 322.39	−712.07	724.58
TC (mg/dL)	2331.8 ± 252.0	2070.7 ± 423.7	−261.08 ± 213.63	213.63	−737.08
HDL-c (mg/dL)	701.8 ± 95.4	709.7 ± 123.1	7.91 ± 66.08	−139.32	155.15
LDL-c (mg/dL)	1405.2 ± 281.1	1136.0 ± 373.5	−269.20 ± 198.84	−712.24	173.84
VLDL-c (mg/dL)	225.2 ± 66.7	225.0 ± 110.8	−0.02 ± 55.98	−124.93	124.53
Creatinine (mg/dL)	9.2 ± 1.0	9.8 ± 1.6	0.57 ± 0.80	−1.20	2.36
Uric acid (mg/dL)	55.7 ± 12.7	56.2 ± 6.1	0.52 ± 5.47	−11.68	12.72
GGT (IU/L)	365.4 ± 400.4	344.4 ± 124.7	−24.04 ± 158.70	−374.64	332.55
ALP (IU/L)	785.6 ± 201.3	859.1 ± 193.9	73.54 ± 115.30	−183.36	330.44
AST (IU/L)	342.8 ± 146.5	266.4 ± 61.7	−76.37 ± 61.02	−212.35	59.61
ALT (IU/L)	263.8 ± 145.9	181.4 ± 50.9	−82.37 ± 58.76	−213.30	48.55
Nitrate (µM)	1155.5 ± 325.7 *	499.9 ± 134.0	−655.66 ± 154.22	−1052.65	−258.67
Nitrite (µM)	24.1 ± 6.9	19.0 ± 3.2	−5.00 ± 3.30	−13.38	3.37
Hemodynamic analysis					
SBP (mmHg)	1535.4 ± 84.3	1443.7 ± 92.9	− 91.68 ± 52.42	−208.49	25.12
DBP (mmHg)	986.0 ± 75.5	896.3 ± 86.1	−89.71 ± 48.03	−196.74	17.31
HR (bpm)	815.8 ± 78.3	740.9 ± 70.5	−74.94 ± 43.16	−171.10	21.22

Abbreviation: ALP, alkaline phosphatase; ALT, alanine transaminase; AST, aspartate transaminase; BMI, body mass index; bpm, beats per minute; DBP, diastolic blood pressure; GGT, γ-glutamyl transpeptidase; HbA1C, glycated hemoglobin; HDL-c, high-density lipoprotein cholesterol; HOMA-B, homeostasis model assessment of beta-cell function; HOMA-IR, homeostatic model assessment of insulin resistance; HR, heart rate; LDL-c, low-density lipoprotein cholesterol; IU/L, international units per liter; kg, kilogram; m, meters; mcIU/dL, micro international unit per milliliter; µM, micromolar; mg/dL, milligrams per deciliter; mmHg, millimeter of mercury; SBP, systolic blood pressure; TC, total cholesterol; TG, triglycerides; VLDL-c, very-low-density lipoprotein cholesterol. Significantly different between BET and PLA groups (* *p* < 0.05). Values are expressed as mean ± standard deviation.

## Data Availability

The original contributions presented in the study are included in the article/Supplementary Material, further inquiries can be directed to the corresponding author.

## Supplementary Materials

The following supporting information can be downloaded at: https://www.mdpi.com/article/10.3390/nu16121942/s1, Table S1: Changes in anthropometric, biochemical, and hemodynamics parameters, show the changes these parameters over the 12-week supplementation period.
