# Supplementary material for: Evaluation of 12-Week Standardized Beetroot Extract Supplementation in Older Participants: A Preliminary Study of Human Health Safety
Source: Nutrients. 2024 Jun 19;16(12):1942. doi: 10.3390/nu16121942 (PMC11206266; doi:10.3390/nu16121942)
Supplement: Supplementary file 1 [file nutrients-16-01942-s001.zip › nutrients-3028896-supplementary.pdf]

Table S1: Changes in anthropometric, biochemical, and hemodynamics parameters, show the changes these parameters over the 12-week supplementation period

	BET					PLA				
	Week 0	Week 4	Week 8	Week 12	AUC	Week 0	Week 4	Week 8	Week 12	AUC
Anthropometric measurements										
Body mass (kg)	68.7 ± 15.8	69.5 ± 16.6	68.8 ± 18.0	69.4 ± 17.8	829.2 ± 205.2	64.0 ± 9.8	63.9 ± 9.1	64.6 ± 9.4	64.8 ± 8.6	771.4 ± 110.2
Height (m)	1.64 ± 0.13	1.64 ± 0.13	1.64 ± 0.13	1.64 ± 0.13	19.20 ± 0.74	1.61 ± 0.07	1.61 ± 0.07	1.61 ± 0.07	1.61 ± 0.07	19.70 ± 1.40
BMI (kg/m^2)	25.4 ± 4.4	25.6 ± 4.2	25.3 ± 4.7	25.6 ± 4.5	305.9 ± 53.3	24.5 ± 2.2	24.5 ± 2.0	24.8 ± 2.1	24.9 ± 1.8	295.8 ± 24.3
Biochemical analysis										
Glucose (mg/dL)	93.4 ± 17.0	103.0 ± 19.1	100.8 ± 22.2	97.2 ± 16.3	1196.4 ± 230.3	103.3 ± 21.2	104.4 ± 25.1	108.8 ± 29.1	105.8 ± 28.8	1271.0 ± 308.3
Insulin (mcUI/mL)	8.0 ± 4.4	7.9 ± 3.6	6.9 ± 3.5	6.0 ± 3.6**	87.3 ± 43.1	6.7 ± 7.0	6.5 ± 6.9	6.5 ± 7.0	6.7 ± 6.9	78.9 ± 82.8
HOMA-IR index	1.9 ± 1.1	2.0 ± 1.0	1.8 ± 1.1	1.5 ± 1.0**	21.9 ± 12.1	2.0 ± 2.6	1.9 ± 2.3	1.9 ± 2.5	2.0 ± 2.5	23.1 ± 29.4
HOMA-B index	102.9 ± 48.7	81.4 ± 40.3	72.9 ±27.2	66.1 ± 32.5*	955.6 ± 405.7	55.8 ± 25.5	57.4 ± 34.3	51.7 ± 28.7	57.9 ± 26.5	664.0 ± 332.0
HbA1C (%)	5.5 ± 0.4	-	-	5.5 ± 0.6	-	5.8 ± 0.9	-	-	5.8 ± 0.9	-
TG (mg/dL)	122.8 ± 62.4	98.8 ± 35.1	107.4 ± 44.4	88.6 ± 31.7	1247.6 ± 386.0	102.4 ± 50.4	106.0 ± 67.0	109.6 ± 48.1	93.2 ± 57.4	1253.9 ± 637.1
TC (mg/dL)	198.0 ± 21.8	189.8 ± 20.2	199.3 ± 23.1	189.7 ± 28.9	2331.8 ± 252.0	171.1 ± 32.3	182.1 ± 45.2	169.3 ± 41.0	161.2 ± 34.1	2070.7 ± 423.7
HDL-c (mg/dL)	60.2 ± 10.7	56.6 ± 8.5	59.3 ± 8.8	58.9 ± 6.1	701.8 ± 95.4	62.1 ± 15.5	61.0 ± 9.3	55.5 ± 7.9	59.6 ± 14.0	709.7 ± 123.1
LDL-c (mg/dL)	116.0 ± 24.3	115.6 ± 23.4	120.4 ± 23.9	114.6 ± 32.1	1405.2 ± 281.1	90.4 ± 32.0	102.1 ± 38.2	94.1 ± 38.2	85.0 ± 27.7	1136.0 ± 373.5
VLDL-c (mg/dL)	22.0 ± 9.8	17.6 ± 6.3	19.6 ± 7.9	16.2 ± 5.7	225.2 ± 66.7	18.6 ± 9.3	19.0 ± 11.5	19.6 ± 8.5	16.6 ± 10.1	225.0 ± 110.8
Creatinine (mg/dL)	0.77 ± 0.12	0.77 ± 0.05	0.77 ± 0.11	0.75 ± 0.09	9.2 ± 1.0	0.81 ± 0.14	0.81 ± 0.09	0.83 ± 0.17	0.84 ± 0.16	9.8 ± 1.6
Uric acid (mg/dL)	4.4 ± 0.9	4.6 ± 1.0	4.7 ± 1.3	4.8 ± 1.2	55.7 ± 12.7	4.8 ± 0.5	4.7 ± 0.5	4.6 ± 0.5	4.7 ± 0.8	56.2 ± 6.1
GGT (UI/L)	28.8 ± 28.1	31.8 ± 37.0	30.3 ± 32.6	29.7 ± 33.0	365.4 ± 400.4	28.3 ± 11.8	29.1 ± 9.9	29.2 ± 10.9	27.2 ± 10.9	344.4 ± 124.7
ALP (UI/L)	66.6 ± 15.7	66.8 ± 19.4	65.6 ± 16.5	61.4 ± 18.3	785.6 ± 201.3	66.3 ± 16.4	71.4 ± 16.2	73.9 ± 18.5	72.7 ± 19.0	859.1 ± 193.9
AST (UI/L)	30.2 ± 17.0	28.8 ± 9.1	27.8 ± 13.3	28.0 ± 13.0	342.8 ± 146.5	23.0 ± 6.3	20.9 ± 4.4	22.6 ± 5.6	23.2 ± 5.6	266.4 ± 61.7
ALT (UI/L)	24.0 ± 16.5	21.2 ± 8.4	20.9 ± 14.0	23.7 ± 13.8	263.8 ± 145.9	14.7 ± 5.3	14.4 ± 3.9	15.9 ± 4.2	15.4 ± 5.6	181.4 ± 50.9
Nitrate (µM)	55.9 ± 50.8	87.0 ± 30.0	124.2 ± 56.3	99.4 ± 65.1	1155.5 ± 325.7†	43.6 ± 18.8	45.4 ± 23.6	42.0 ± 17.8	31.4 ± 7.3	499.9 ± 134.0
Nitrite (µM)	1.5 ± 0.3	1.7 ± 0.4	3.0 ± 1.9	1.8 ± 0.5	24.1 ± 6.9	1.5 ± 0.3	1.6 ± 0.3	1.8 ± 0.4	1.4 ± 0.2	19.0 ± 3.2
Hemodynamic analysis										
SBP (mmHg)	126.8 ± 6.3	125.2 ± 7.9	130.5 ± 9.3	129.5 ± 13.0	1535.4 ± 84.3	119.9 ± 7.8	120.7 ± 8.8	121.4 ± 12.7	117.9 ± 8.5	1143.7 ± 92.9
DBP (mmHg)	86.0 ± 11.1	79.6 ± 4.6	82.4 ± 7.6	83.0 ± 8.2	986.0 ± 75.5	74.4 ± 7.5	74.9 ± 6.9	74.6 ± 7.8	74.9 ± 8.5	896.3 ± 86.1
HR (bpm)	73.8 ± 10.4	65.4 ± 5.9	68.3 ± 8.6	66.7 ± 7.4	815.8 ± 78.3	64.7 ± 10.6	60.6 ± 7.2	61.4 ± 5.4	61.7 ± 7.1	740.9 ± 70.5

Abbreviation: ALP, alkaline phosphatase; ALT, alanine transaminase; AST, aspartate transaminase; BMI, body mass index; bpm, beats per minute; DBP, diastolic blood pressure; GGT, γ-glutamyl transpeptidase; HbA1C, glycated hemoglobin; HDL-c, high-density lipoprotein cholesterol; HOMA-B, homeostasis model assessment of beta-cell function; HOMA-IR, homeostatic model assessment of insulin resistance; HR, heart rate; LDL-c, low-density lipoprotein cholesterol; IU/L, international units per liter; kg, kilogram; m, meters; mcIU/dL, micro international unit per milliliter; µM, micromolar; mg/dL, milligrams per deciliter; mmHg, millimeter of mercury; SBP, systolic blood pressure; TC, total cholesterol; TG, triglycerides; VLDL-c, very- low-density lipoprotein cholesterol. Significantly different from week 0 (* $p < 0.05$), week 4 (** $p < 0.05$) within group BET, and between groups († $p < 0.05$). Values were expressed as mean ± standard deviation.
